# The Eye Never Lies: When A Lesion Unveils Lung Cancer

**DOI:** 10.1002/rcr2.70540

**Published:** 2026-03-11

**Authors:** Sivanesan Darshini, Nor Safiqah Sharil, Boon Hau Ng, Xin Yee Chong, Andrea Yu‐Lin Ban, Nik Nuratiqah Nik Abeed

**Affiliations:** ^1^ Respiratory Unit, Department of Medicine, Faculty of Medicine, Hospital Canselor Tuanku Muhriz Universiti Kebangsaan Malaysia Kuala Lumpur Malaysia; ^2^ Internal Medical Unit, Faculty of Medicine and Health Science Universiti Sains Islam Malaysia Nilai Negeri Sembilan Malaysia; ^3^ Ophthalmology Department Hospital Cyberjaya Selangor Malaysia

**Keywords:** iris metastasis, lung adenocarcinoma, pembrolizumab

## Abstract

Ocular manifestations rarely represent the first sign of systemic malignancy and often pose a diagnostic challenge. While choroidal metastases are more common, iris involvement is uncommon and may be underrecognized. Lung adenocarcinoma, particularly non‐small cell subtypes, is a known source of ocular metastases, with iris lesions occasionally preceding systemic symptoms. We report a 70‐year‐old previously healthy man presenting with one month of left eye blurred vision. Initial examination revealed conjunctival injection and visual acuity of 6/20 without detectable lesions; topical dexamethasone was prescribed. Six weeks later, vision declined to finger counting, and a hypopigmented iris mass (7.0 × 5.7 mm) was identified. A brain MRI revealed a right occipital lesion, and a CECT of the thorax, abdomen, and pelvis showed a left lung mass with bilateral nodules, mediastinal lymphadenopathy, and right iliac metastasis. CT‐guided biopsy confirmed stage IV lung adenocarcinoma (TTF‐1 and Napsin A positive, PD‐L1 TPS 80%, EGFR/ALK/ROS1 negative). Pembrolizumab was initiated; filamentous keratitis developed during therapy, and progression at three months prompted conventional chemotherapy. This case emphasizes the rarity of iris metastasis as the initial presentation of lung adenocarcinoma.

## Graphical abstract

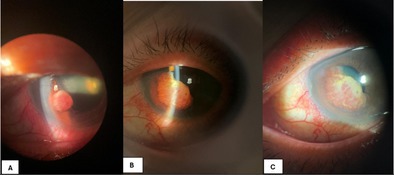

## 1 Introduction

Ocular metastases are rare, pose a diagnostic challenge, and can be the first sign of an underlying tumour. The incidence of ocular metastasis has been reported to be as high as 8%–10%. Among metastatic eye lesions, the majority originate from breast cancer (47%) and lung cancer (21%). The uvea is the most common site of metastasis due to its rich vascular supply; in contrast, iris lesions are rare but easily visualized. Lung adenocarcinoma, particularly non‐small cell subtypes, has been identified as a frequent source of ocular metastases, accounting for up to 90% of cases in some studies [1]. We present a rare case of asymptomatic primary lung adenocarcinoma that first manifested with ocular symptoms and an iris lesion.

## 2 Case Report

**FIGURE 1:**
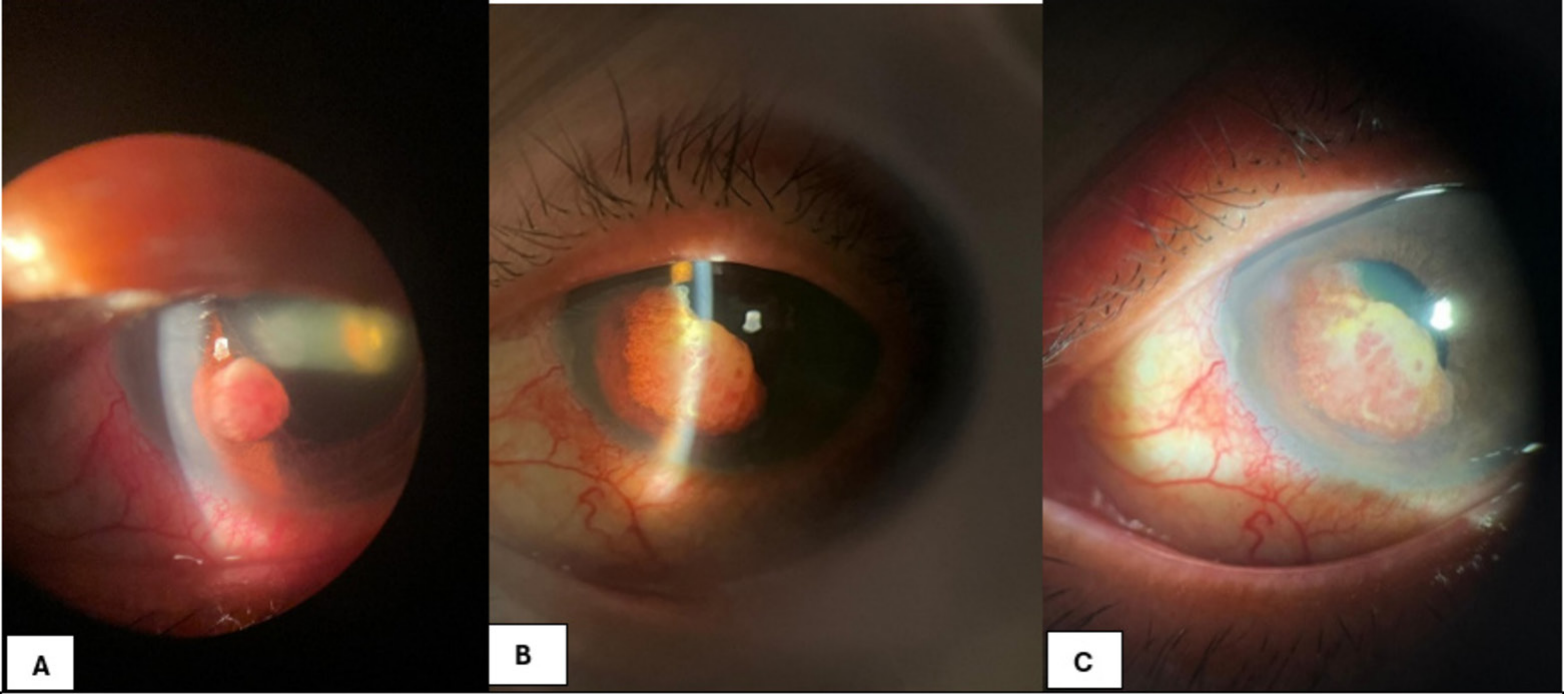
(A) Iris mass seen at anterior chamber of the left eye at Week 2. (B) Larger iris mass of the left eye at the week 6 assessment.

A 70‐year‐old never‐smoker with no underlying medical comorbidities and an ECOG performance status of 0 presented with 1 month of left eye blurred vision. Initial assessment revealed a red eye and visual acuity of 6/20 with no identifiable lesions. He was treated with topical dexamethasone. At the six‐week follow‐up, vision had deteriorated to finger counting. An examination revealed a hypopigmented iris mass measuring 7.0 mm × 5.7 mm, suspicious for iris metastasis, with amelanotic iris melanoma as a differential diagnosis, prompting a systemic workup (Figure 1). A biopsy was not performed at that stage. The tumour markers were sent; carcinoembryonic antigen (CEA) was markedly elevated at 85 ng/mL, while alpha‐fetoprotein (AFP) and CA19‐9 were within normal limits.

**FIGURE 2:**
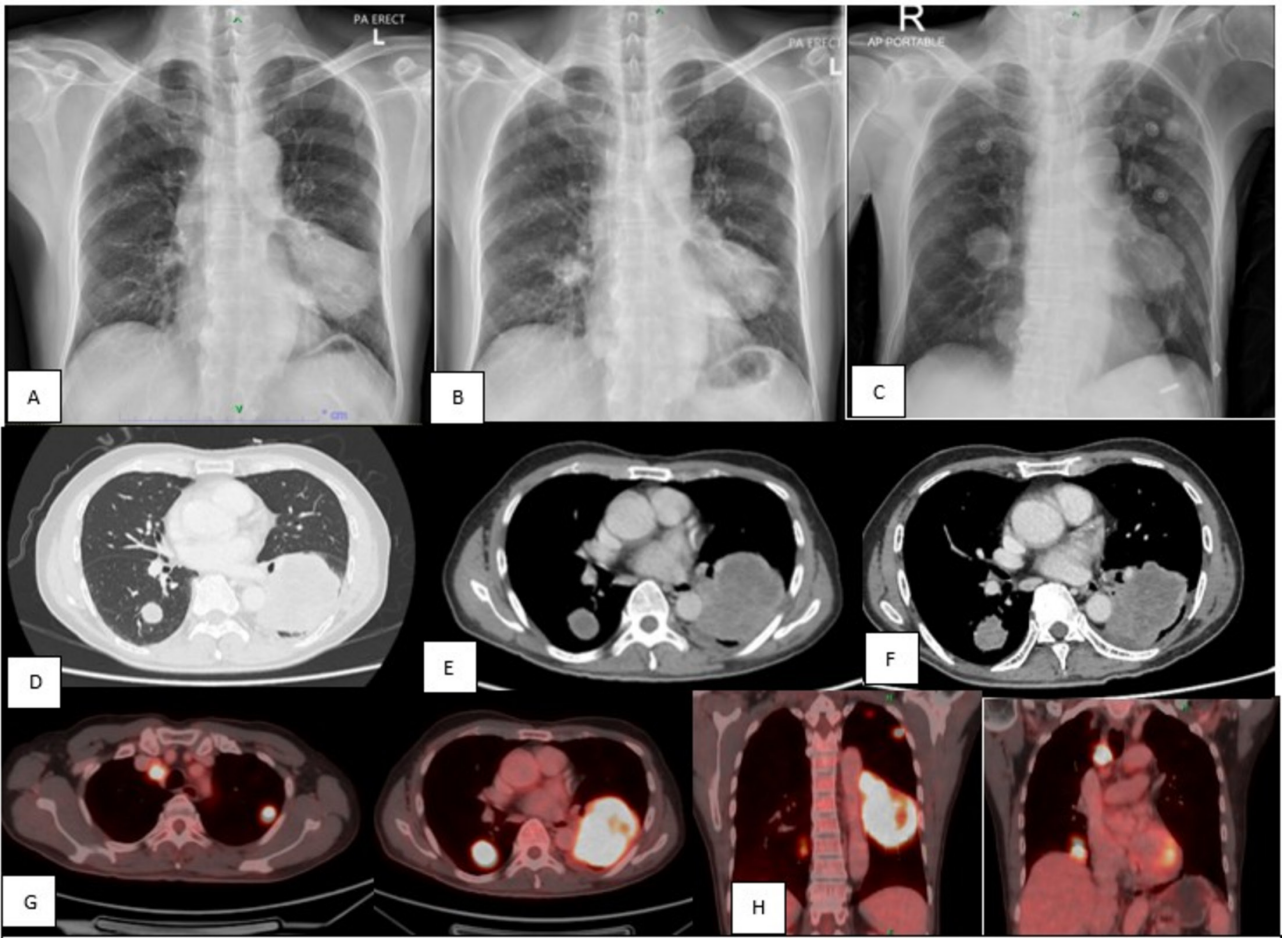
(A–C) Chest radiograph showing left lower zone mass and enlarging mass with multiple nodular opacities in bilateral lungs at diagnosis, 8 weeks and 12 weeks immunotherapy. (D–F) CECT thorax showing an irregular mass at the left lower lobe and a nodule at the left lower lobe with an enlarging mass and multiple nodular metastases at diagnosis, 8 and 12 weeks of immunotherapy. G and H showed PET CT at 12 weeks post‐immunotherapy.

Contrast‐enhanced computed tomography (CECT) of the brain and orbits showed no detectable lesions; however, magnetic resonance imaging (MRI) of the brain revealed a right intra‐axial occipital lesion, in addition to a left iris lesion. The chest radiography and CECT of the thorax, abdomen and pelvis demonstrated a left lung mass measuring 6.3 × 5.7 × 8.3 cm (AP × W × CC), associated with bilateral pulmonary nodules, mediastinal lymphadenopathy and a right iliac bone lesion (Figure 2). A CT‐guided biopsy of the left lower lobe lung mass was performed, and histopathological examination confirmed lung adenocarcinoma, with immunohistochemistry positive for thyroid transcription factor‐1 (TTF‐1) and Napsin A. The molecular studies were negative for EGFR mutations, ALK fusion, and ROS1 rearrangement, with a programmed death‐ligand 1 (PD‐L1) tumour proportion score of 80%. The patient was diagnosed with stage IV lung adenocarcinoma (T4N3M1b).

He was treated with pembrolizumab 100 mg every 3 weeks. Both the right occipital and left iris lesions were amenable to gamma knife stereotactic radiosurgery; however, the patient declined the procedure. During the course of immunotherapy, follow‐up evaluation of the iris metastasis demonstrated a stable lesion; however, there was a new development of filamentary keratitis in the left eye, requiring treatment with topical antibiotic and steroid eye drops. Repeat CT imaging at 8 and 12 weeks after initiation of immunotherapy demonstrated interval enlargement of the pulmonary and mediastinal metastatic lesions, consistent with disease progression. Conventional chemotherapy was commenced.

## 3 Discussion

Intraocular metastases most commonly originate from breast and lung cancers and are far more frequent than primary iris melanoma, often with associated CNS involvement. Iris metastasis is rare, accounting for approximately 9% of cases compared with 88%–89% involving the choroid, and may mimic amelanotic melanoma. Although histopathological confirmation is ideal, iris biopsy is seldom performed due to procedural risks, and diagnosis is usually inferred from systemic evaluation. In some cases, ocular symptoms may precede the diagnosis of lung cancer, posing a significant diagnostic challenge.

At initial presentation, the patient lacked typical features of iris metastasis, such as blurred vision, ocular pain, redness, or a visible iris mass, which only became evident during subsequent follow‐up. A rapidly enlarging iris lesion, particularly when accompanied by constitutional symptoms, should raise suspicion for underlying systemic malignancy. Although fine‐needle aspiration biopsy is recommended for cytological differentiation, with Tao Liu et al. reporting a diagnostic yield of 49% using fine‐needle aspiration, iris biopsy, or enucleation, a lung biopsy was more feasible in our case and provided a definitive diagnosis.

The literature on iris metastasis is limited and consists largely of isolated case reports describing iris lesions as the initial manifestation of malignancy involving vital organs, with lung adenocarcinoma being particularly rare. Table 1 demonstrates the heterogeneity of lung malignancies presenting as iris metastasis. While various histological subtypes have been described, adenocarcinoma presenting initially as iris metastasis remains particularly rare.

**TABLE 1:** Reported cases of iris metastasis as initial presentation of lung malignancy.

Author	Age/sex	Iris findings	Diagnostic modality	Final diagnosis	Remarks
Ganesan et al. [2]	65/M	Small, well‐circumscribed amelanotic iris mass	Iris biopsy; systemic imaging	Poorly differentiated lung adenocarcinoma	Iris lesion was the first manifestation
Kelly et al. [3]	60/M	Exudative iris mass	Systemic imaging	Lung adenocarcinoma with adrenal metastases	Multiple pulmonary nodules detected
Mukaddes et al. [4]	46/M	Bilateral vascularized iris masses	Supraclavicular lymph node biopsy	Small cell lung carcinoma	Bilateral ocular involvement
Hirakata and Nakao [5]	70/M	Neovascularized iris mass in anterior chamber	Histopathology	Lung squamous cell carcinoma	Non‐adenocarcinoma subtype

## Author Contributions

Darshini conceptualized the study, drafted the manuscript, and contributed to data collection. Ng Boon Hau and Nor Safiqah Sharil performed clinical evaluation of the patient and provided critical revisions to the manuscript. Xin Yee Chong conducted radiological and pathological review, interpreted imaging and biopsy results, and contributed to discussion writing. Andrea Yu‐Lin Ban and Dr. Nik Nuratiqah Nik Abeed reviewed literature, prepared figures and tables, and assisted in manuscript editing.

## Funding

The authors have nothing to report.

## Consent

The authors declare that written informed consent was obtained for the publication of this manuscript and accompanying images using the consent form provided by the journal.

## Conflicts of Interest

Andrea Yu‐Lin Ban is an Editorial Board member of Respirology Case Reports and a co‐author of this article. She was excluded from all editorial decision‐making related to the acceptance of this article for publication. The other authors declare no conflicts of interest.

## Data Availability

The data that support the findings of this study are available from the corresponding author upon reasonable request.
